# The relative importance of work-related psychosocial factors in physician burnout

**DOI:** 10.1093/occmed/kqab147

**Published:** 2021-11-03

**Authors:** K Gluschkoff, J J Hakanen, M Elovainio, J Vänskä, T Heponiemi

**Affiliations:** 1 Finnish Institute for Health and Welfare, Helsinki, Finland; 2 Department of Psychology and Logopedics, University of Helsinki, Helsinki, Finland; 3 Finnish Institute of Occupational Health, Helsinki, Finland; 4 Finnish Medical Association, Helsinki, Finland

**Keywords:** Burnout, dominance analysis, job demands, job resources, physicians

## Abstract

**Background:**

Identifying the most significant risk factors for physician burnout can help to define the priority areas for burnout prevention. However, not much is known about the relative importance of these risk factors.

**Aims:**

This study was aimed to examine the relative importance of multiple work-related psychosocial factors in predicting burnout dimensions among physicians.

**Methods:**

In a cross-sectional sample of 2423 Finnish physicians, dominance analysis was used to estimate the proportionate contribution of psychosocial factors to emotional exhaustion, depersonalization and reduced personal accomplishment. The psychosocial factors included job demands (time pressure, patient-related stress, lack of support, stress related to information systems, work–family conflict) and job resources (job control, team climate, organizational justice).

**Results:**

Together, psychosocial factors explained 50% of the variance in emotional exhaustion, 24% in depersonalization and 11% in reduced professional efficacy. Time pressure was the most important predictor of emotional exhaustion (change in total variance explained Δ*R*^2^ = 45%), and patient-related stress was the most important predictor of both depersonalization (Δ*R*^2^ = 52%) and reduced professional accomplishment (Δ*R*^2^ = 23%). Stress related to information systems was the least important predictor of the burnout dimensions (Δ*R*^2^ = 1–2%).

**Conclusions:**

Psychosocial factors in physicians’ work are differently associated with the dimensions of burnout. Among the factors, the most significant correlates of burnout are job demands in the form of time pressure and patient-related stress.

**Key learning points**

**What is already known about this subject:**

- Burnout is a prevalent occupational hazard among physicians.
- Developing effective burnout interventions requires an understanding of the key work-related psychosocial risk factors for burnout.
- The relative importance of these risk factors is unclear because previous studies have typically focused on a single or a few potential determinants of physician burnout.

**What this study adds:**

- In a large nationally representative sample of Finnish physicians, this cross-sectional study used dominance analysis to examine the proportionate contribution of several psychosocial factors to the three burnout dimensions: emotional exhaustion, depersonalization and reduced personal accomplishment.
- Time pressure was the most important predictor of emotional exhaustion and patient-related stress was the most important predictor of both depersonalization and reduced professional accomplishment.
- Stress related to information systems was the least important predictor of the burnout dimensions.

**What impact this may have on practice or policy:**

- Reducing job demands such as time pressure and patient-related stress may be a useful strategy to protect physicians from burnout.

## Introduction

Burnout is a prevalent occupational hazard among physicians [1] that includes feelings of emotional exhaustion, depersonalization and reduced personal accomplishment [2]. Emotional exhaustion refers to feelings of being overextended and depleted of emotional and physical resources; depersonalization to negative, cynical or detached response to patients; and reduced personal accomplishment refers to feelings of incompetence and inefficiency at work [3]. Burnout has serious consequences for physician’s health and quality of life, including mental health problems, sickness absence and suicidality [4,5]. Physician burnout also compromises the quality of patient care by increasing the risk of medical errors, workplace violence and turnover intention [6].

The work-related psychosocial risk factors for burnout—such as time pressure, lack of workplace support, poor job control and organizational injustice [6,7]—can be categorized into high job demands and low job resources [8]. There is considerable evidence on the antecedents of burnout from studies examining emotional exhaustion, the core dimension of burnout that is suggested to represent its first stage of development [9]. Less attention has been paid to the work-related antecedents of depersonalization and reduced personal accomplishment. With regard to physician burnout, new risk factors for burnout may have emerged during the past decades as a consequence of changes in physicians’ work environment and developments in technology. For example, emerging evidence suggests that working with poorly functioning, difficult to use health information systems may contribute to higher burnout rates among physicians [10,11].

Developing effective burnout interventions for physicians requires an understanding of the key work-related psychosocial risk factors for burnout. However, the relative importance of these risk factors is unclear because previous studies have typically focused on a single or a few potential determinants of burnout among physicians. The present study addressed this gap in the literature by using dominance analysis to compare the relative importance of several work-related psychosocial factors to physician burnout in a large nationally representative sample of Finnish physicians. Whereas traditional regression techniques are not suitable for this purpose [12], dominance analysis allows researchers to examine the contribution of multiple correlated predictors to an outcome [13,14]. To provide a comprehensive understanding of the potential work-related psychosocial antecedents of burnout, we considered several job demands and resources that reflected both established, so-called challenge demands (e.g. time pressure) and newer risk factors, so-called hindrance demands (e.g. stress related to information systems) as well as lack of salient job resources (e.g. lacking social support) for burnout among physicians.

## Methods

We used cross-sectional data collected in 2019 as part of the ongoing Finnish Health Care Professionals Study (HPS) launched in 2006. A random, nationally representative sample of physicians was drawn from a database maintained by the Finnish Medical Association including all active licensed physicians in Finland. The sample members received an e-mail invitation to participate in a web-based survey, followed by two reminders. A postal questionnaire was sent once to those who did not respond. A total of 3513 responses were received (response rate 44%). For the present study, respondents who were not participating in working life (e.g. due to retirement or family leave, *n* = 569) or who were older than 65 years old (*n* = 42) were excluded. Those with missing data were also excluded (*n* = 479), resulting in a final sample size of *N* = 2423 (for the pattern of missingness, see Figure S1, available as Supplementary data at *Occupational Medicine* Online). Ethical approval for HPS was obtained from the ethics committee of the National Research and Development Centre for Welfare and Health in Finland.

Burnout was measured with the 22-item Maslach Burnout Inventory—Human Services Survey for Medical Personnel (MBI-HSS (MP) [3]). MBI-HSS (MP) measures three dimensions of burnout: emotional exhaustion (nine items, e.g. ‘I feel tired when I get up in the morning and have to face another day on the job’), depersonalization (five items, e.g. ‘I feel I treat some patients as if they were impersonal objects’) and reduced personal accomplishment (eight items, e.g. ‘I deal very effectively with the problems of my patients’ [reversed]). The items were rated on a 7-point scale (0 = never, 1 = a few times a year, 2 = once a month, 3 = a few times a month, 4 = once a week, 5 = a few times a week and 6 = everyday). The scores for each dimension were summed to obtain a total score, higher scores representing more severe emotional exhaustion, depersonalization and reduced personal accomplishment.

Psychosocial factors included job demands (time pressure, patient-related stress, lack of support, stress related to information systems and work–family conflict) and job resources (job control, team climate and organizational justice). Job demands other than work–family conflict were measured with items asking how often during the past 6 months the respondent had been distracted by, worried about or stressed about *time pressure* (three items, e.g. ‘Too little time to do work properly’); *patients* (three items, e.g. ‘Difficult patients who complain, blame or criticize’); *lack of support* (three items, e.g. ‘Lack of consultation possibilities’); and poorly functioning, constantly changing *information systems* (two items, e.g. ‘difficult, poorly functioning information systems or applications’). The items were rated on a 5-point scale, ranging from 1 = rarely or never to 5 = very often or constantly. *Work–family conflict* was measured with a scale developed by Frone *et al*. [15] asking how often the respondent’s job interfered with their family life (two items, e.g. How often your job or career interferes with your responsibilities at home, such as cooking, shopping, child care, home maintenance and repairs?”), rated on a 5-point scale, ranging from 1 = never to 5 = very often. *Job control* was measured with items from Karasek’s Job Content Questionnaire [16] that measure decision authority (three items, e.g. ‘My job allows me to make a lot of decisions on my own’). *Team climate* was measured with Team Climate Inventory’s [17] participative safety subscale that measures interpersonal atmosphere and information sharing (four items, e.g. ‘People feel understood and accepted by each other’). *Organizational justice* was measured with the short version [18] of a justice scale originally developed by Colquitt [19] (eight items, e.g. ‘Everybody is entitled to express their opinions and views in matters that concern them’). The items measuring job control, team climate and organizational justice were rated on a 5-point scale, ranging from 1 = totally disagree to 5 = totally agree. Mean scores were calculated for each psychosocial work characteristics variable. For a full list of the items, see Table S1 (available as Supplementary data at *Occupational Medicine* Online).

As an initial step, we calculated Cronbach alphas (measure of internal consistency that is used to estimate the reliability of a scale) for each variable and examined bivariate correlations between the variables. For the main analysis, dominance analysis [13,14] was performed to examine the relative importance of psychosocial factors in predicting exhaustion, depersonalization and reduced personal accomplishment (each outcome separately). The method can be used to determine which predictors are most important in terms of their proportional contribution to the explained variance in an outcome [13]. Whereas interpreting regression coefficients from a traditional multiple regression can lead to incorrect conclusions about predictor importance when the predictors are correlated (i.e. are multicollinear) [14], dominance analysis is robust to multicollinearity. The method computes the change in variance explained (Δ*R*^2^) from adding a specific predictor to all possible subset regression models and then averages the Δ*R*^2^ across all subsets for each predictor. The approach does not involve any assumptions about the causal order of the predictors. The analyses and data visualizations were conducted in R Statistical Software version 3.6.1 using the packages yhat [20] and ggplot2 [21].

## Results

The sample size was 2423. Average age was 44.66 years (SD = 11.19), 67% were female and the majority of respondents (77%) worked in the public sector. The prevalence of burnout in the sample was 37%, as determined by the convention to define burnout as high emotional exhaustion (a score of ≥27) or high depersonalization (a score of ≥10) [22,23].

Descriptive statistics including Cronbach alphas and Pearson correlations for the study variables are presented in Table 1. Several work-related psychosocial factors were moderately to strongly correlated with each other, which supported our decision to use dominance analysis as the main modelling strategy to examine their combined associations with burnout. Time pressure and patient-related stress were among the strongest correlates of exhaustion and depersonalization. In addition, lack of support, lack of job control and work–family conflict were rather strongly correlated with exhaustion. Although several psychosocial factors were also correlated with reduced personal accomplishment, in general, the correlations were weaker in comparison to the correlations between psychosocial factor and exhaustion or depersonalization. Depersonalization and reduced personal accomplishment were more strongly correlated with emotional exhaustion than with any work-related psychosocial factor.

**Table 1.** Descriptive statistics and Pearson correlations between the study variables (Cronbach alphas on the diagonal)

	Variable	Range	*M* (SD)	1	2	3	4	5	6	7	8	9	10	11
	Job demands													
1.	Time pressure	1–5	3.40 (1.01)	(0.86)										
2.	Patient-related stress	1–5	2.50 (0.81)	**0.30**	(0.82)									
3.	Lack of support	1–5	2.30 (0.80)	**0.40**	0.27	(0.62)								
4.	Stress related to information systems	1–5	3.37 (1.04)	**0.31**	0.18	0.25	(0.75)							
5.	Work–family conflict	1–5	3.26 (1.07)	**0.42**	0.08	0.19	0.14	(0.85)						
	Job resources													
6.	Job control	1–5	3.95 (0.80)	**−0.35**	−0.16	**−0.33**	−0.18	−0.22	(0.74)					
7.	Team climate	1–5	3.86 (0.82)	−0.18	−0.07	**−0.58**	−0.14	−0.13	**0.35**	(0.89)				
8.	Organizational justice	1–5	3.78 (0.78)	−0.28	−0.07	**−0.53**	−0.21	−0.22	**0.45**	**0.61**	(0.87)			
	Burnout													
9.	Emotional exhaustion	0–54	19.82 (11.95)	**0.64**	**0.35**	**0.42**	0.21	**0.42**	**−0.36**	−0.26	**−0.30**	(0.92)		
10.	Depersonalization	0–30	5.72 (5.43)	**0.30**	**0.41**	0.25	0.12	0.20	−0.26	−0.18	−0.16	**0.51**	(0.76)	
11.	Reduced personal accomplishment	0–47	8.39 (6.83)	0.20	0.20	0.22	0.08	0.11	−0.22	−0.22	−0.19	**0.32**	0.29	(0.81)

Correlations >|0.03| are significant at *P* <0.05. Moderate-to-strong (*r* > |0.30|) correlations are shown in boldface. Cronbach alphas are shown on the diagonal in parenthesis.

**Figure 1.**
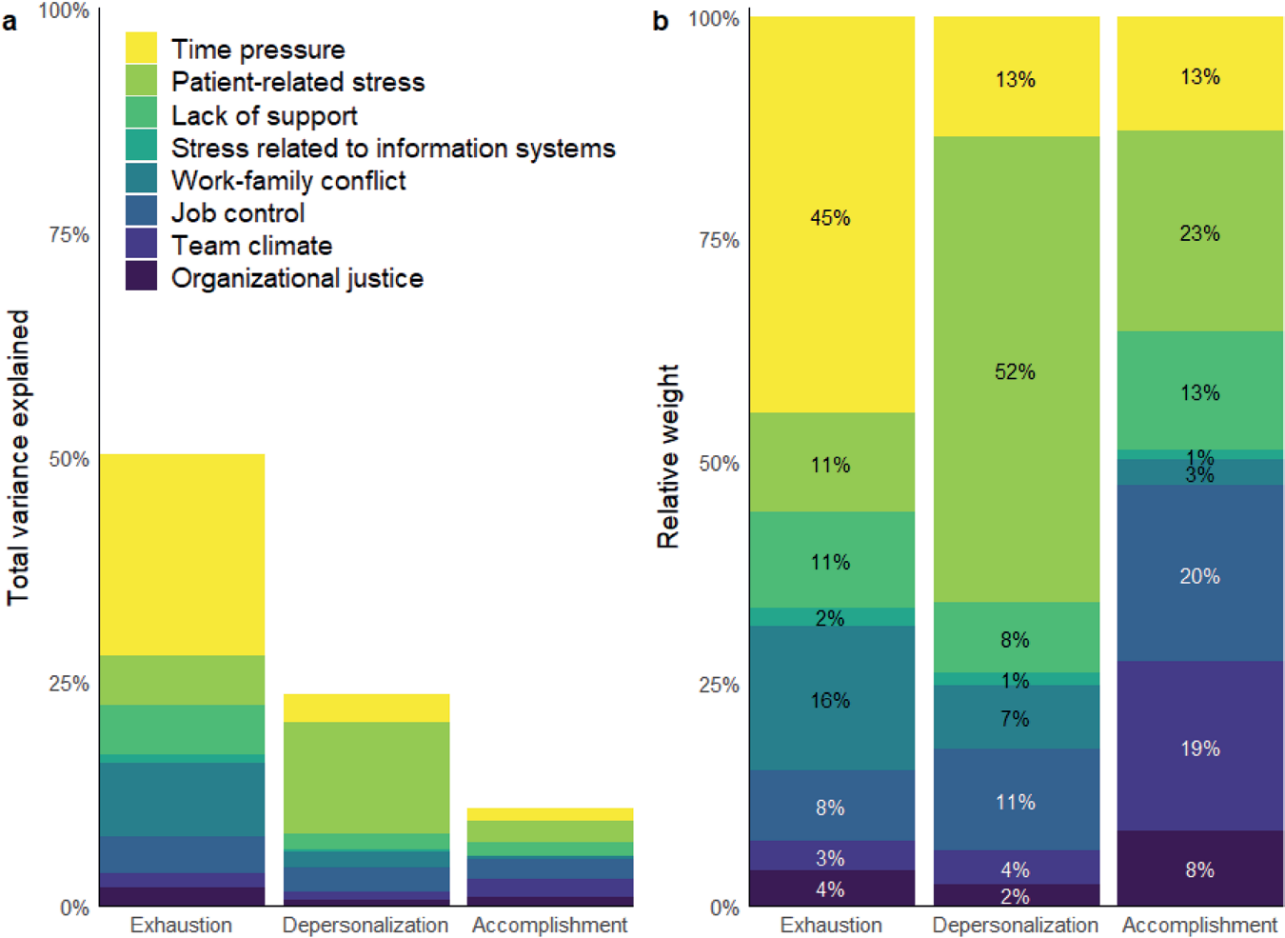
The results of dominance analysis. (a) The importance of work-related psychosocial factors in predicting the dimensions of burnout as raw relative weights. (b) The relative weights are scaled to show the percent of variance explained. Due to rounding, the percentages may not add up to 100%.

The results of dominance analysis are shown in Figure 1. Together, work-related psychosocial factors explained 50% of variance in emotional exhaustion, 24% in depersonalization and 11% in reduced personal accomplishment. As for the relative importance of psychosocial factors, around half of the total explained variance (*R*^2^) in emotional exhaustion and depersonalization was attributable either to time pressure or to patient-related stress. Time pressure (change in variance explained Δ*R*^2^ = 45%), work–family conflict (Δ*R*^2^ = 16%), patient-related stress (Δ*R*^2^ = 11%) and lack of support (Δ*R*^2^ = 11%) were the most important predictors of emotional exhaustion (each accounting for at least 10% of the total variance explained); patient-related stress (Δ*R*^2^ = 52%), time pressure (Δ*R*^2^ = 13%) and poor job control (Δ*R*^2^ = 11%) were the most important predictors of depersonalization; and patient-related stress (Δ*R*^2^ = 23%), poor job control (Δ*R*^2^ = 20%), poor team climate (Δ*R*^2^ = 19%), time pressure (Δ*R*^2^ = 13%) and lack of support (Δ*R*^2^ = 13%) were the most important predictors of reduced personal accomplishment. Stress-related to information systems was the least important predictor, contributing only from 1 to 2% to the total variance explained.

If totalled, psychosocial factors reflecting job demands (time pressure, patient-related stress, lack of support, stress related to information systems and work–family conflict) accounted for almost all (from 81 to 85%) of the total variance explained in emotional exhaustion and depersonalization (Figure 1b). The explained variance in reduced personal accomplishment was more dispersed between job demands and job resources.

## Discussion

Considered together, psychosocial factors at work were strongly associated with the emotional exhaustion dimension of burnout and, to a lesser degree, with depersonalization and reduced personal accomplishment. For emotional exhaustion, the most important predictors were (in order of importance) time pressure, work–family conflict, patient-related stress and lack of support; for depersonalization, patient-related stress, time pressure and poor job control; and for reduced personal accomplishment, patient-related stress, poor job control, poor team climate, time pressure, lack of support and organizational injustice. Overall, particularly time pressure and patient-related stress emerged as important predictors of physician burnout, accounting for around half of the total variance explained in emotional exhaustion and depersonalization, respectively.

A major strength of the study is that we examined the dimensions of burnout instead of the total burnout score or a binary variable for burnout caseness. Because the three dimensions are distinct constructs [2], combining them to form an overall burnout score could obscure the associations of interest. Other strengths of the study are its relatively large sample size and the inclusion of several established and newer risk factors as potential predictors of physician burnout. However, several limitations need to be considered when interpreting the results. The study was based on cross-sectional data, which does not allow drawing causal inferences. The cross-sectional design is susceptible to reverse causality, meaning that having burnout symptoms could lead to perceptions of high job demands and low job resources. Because we used self-report measures, there is a possibility that common method variance inflated the observed associations. The data included only a few measures of job resources. For example, support was measured as a job demand (i.e. lack of support) rather than as a job resource due to the wording of the questionnaire. Furthermore, the reliability of the lack of support scale was low, below the conventional cut-off of 0.7 for Cronbach alpha. The use of complete case analysis may have introduced bias in the results. Finally, it should be noted that the results of dominance analysis depend on the set of predictor variables included in the analysis [13]. The relative importance of the psychosocial factors could therefore be different if additional factors were considered. It should also be noted that physicians’ work has undergone significant changes in the past years due to the COVID-19 pandemic. For example, the increase in remote consultations has probably changed patient interactions in primary care. The pandemic may have also introduced new stressors into the workplace that threaten physicians’ mental well-being, which could be explored in future studies.

Our findings are in line with previous research linking excessive workload and time pressure with emotional exhaustion among physicians and also in other professions [2,6,7,23]. Considering that emotional exhaustion forms the core of burnout, our results highlight the significance of time pressure as an important work-related factor associated with burnout among physicians. The results also extend previous research by demonstrating the importance of patient-related stress in predicting physician depersonalization and reduced accomplishment.

Stress-related to information systems was the least important predictor of the burnout dimensions, which contrasts with the results of some previous studies that suggest an association with the use of health information technology and higher burnout among physicians [10,11]. However, in contrast to the current study, previous studies linking health information system use and burnout have not examined the importance of several psychosocial factors in physicians’ work environment. Even though stress related to information systems and physician burnout may be associated, our results indicate that the association is rather trivial in comparison to that of other work-related psychosocial factors.

Considered together, psychosocial factors reflecting job demands in physicians’ work accounted for much of the associations with emotional exhaustion and depersonalization. With regard to reduced personal accomplishment, the associations were more equally attributable to both job demands and job resources. Our findings align with the conservation of resources (COR) theory [9], which has been widely applied to explain the development of burnout and has received empirical support from many previous studies [24–26]. The theory suggests that people are more sensitive to demands placed on them (resource loss) than to the resources they received (resource gain) [27]. According to the theory, job demands, rather than job resources, are strongly associated particularly with the emotional exhaustion dimension of burnout. In the current study, job demands were strongly associated with both exhaustion and depersonalization.

Our results are also in agreement with the view that reduced personal accomplishment may develop as a consequence of exhaustion and depersonalization rather than directly from work-related factors [2,28]. Work-related psychosocial factors explained only a relatively small amount of variance in reduced personal accomplishment. Furthermore, reduced personal accomplishment was more strongly correlated with exhaustion and depersonalization than with any of the work-related psychosocial factors. The results imply that in identifying the potential modifiable risk factors for physician burnout in the workplace, determining the work-related correlates of exhaustion and depersonalization may be most useful. It is worth noting that as a result of accumulated evidence on the divergent and minor role of personal accomplishment in burnout [29], in more recent burnout instruments, this dimension has been removed (e.g. Oldenburg Burnout Inventory) or replaced by more recognized burnout symptoms of cognitive and emotional impairment (e.g. Burnout Assessment Tool).

In conclusion, we found that different job demands, particularly time pressure and patient-related stress, are likely to be the breeding ground for physician burnout. Approximately 85% of the total variance explained in emotional exhaustion and depersonalization was accounted by various job demands, whereas the remaining 15% was accounted by (lack of) various job resources, such as poor job control and poor team climate. This suggests that reducing job demands may be a useful strategy to protect physicians from burnout. However, job demands are relatively inherent to the situation and workplace and, therefore, difficult to change. Job resources, in turn, represent more alterable job characteristics [30]. Therefore, in the shorter term, it might be useful to focus on enhancing various job resources (e.g. colleague and supervisor support) in physicians’ work and in the longer term, also on building professional resources, such as skills and collaborative practices in meeting difficult patient situations. By increasing job resources, it would be possible to also mitigate the negative impacts of various job demands.

## Supplementary Material

kqab147_suppl_Supplementary_Material
