# Supplementary material for: The relative importance of work-related psychosocial factors in physician burnout
Source: Occup Med (Lond). 2021 Nov 3;72(1):28–33. doi: 10.1093/occmed/kqab147 (PMC8758190; doi:10.1093/occmed/kqab147)
Supplement: kqab147_suppl_Supplementary_Material [file kqab147_suppl_supplementary_material.docx]

**The relative importance of work-related psychosocial factors in physician burnout**

**Supplementary material**


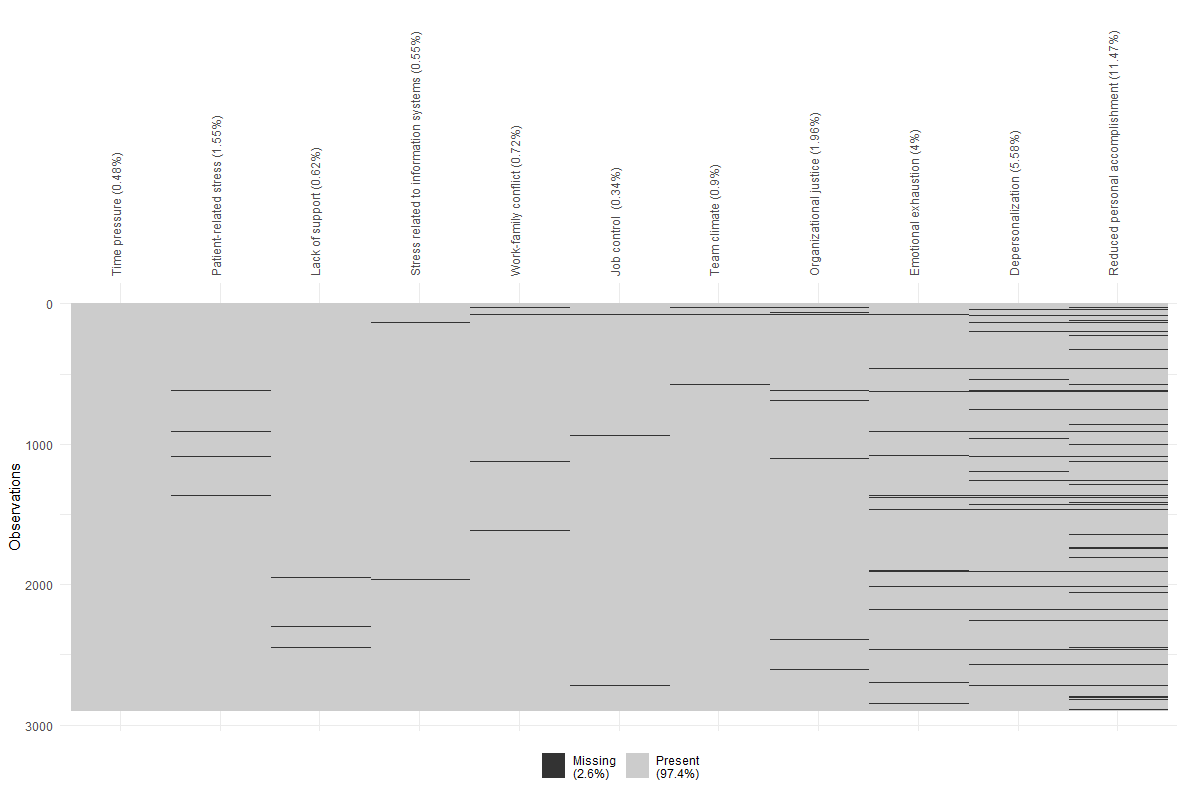


**Supplementary Figure S1.** The pattern of missingness in the study variables.

**Supplementary Table S1.** Questionnaire items that were used to measure work-related psychosocial factors

| **Job demands** | | | |
| --- | --- | --- | --- |
| Time pressure | How often during the past 6 months have you been distracted by, worried about or stressed about the following issue: | Constant rush and pressure due to uncompleted work. | |
|  | How often during the past 6 months have you been distracted by, worried about or stressed about the following issue: | Too little time to do work properly | |
|  | How often during the past 6 months have you been distracted by, worried about or stressed about the following issue: | Tight and inflexible work pace | |
| Patient-related stress | How often during the past 6 months have you been distracted by, worried about or stressed about the following issue: | Difficult patients who complain, blame, or criticize | |
|  | How often during the past 6 months have you been distracted by, worried about or stressed about the following issue: | Patients’ expectations frequently differ from those of health care personnel | |
|  | How often during the past 6 months have you been distracted by, worried about or stressed about the following issue: | Patients are unwilling to co-operate, and they are passive | |
| Lack of support | How often during the past 6 months have you been distracted by, worried about or stressed about the following issue: | Lack of consultation possibilities | |
|  | How often during the past 6 months have you been distracted by, worried about or stressed about the following issue: | Insufficient co-operation in the work group | |
|  | How often during the past 6 months have you been distracted by, worried about or stressed about the following issue: | Lack of trust and openness in workplace | |
| Stress related to information systems | How often during the past 6 months have you been distracted by, worried about or stressed about the following issue: | Changing electronic health-record systems | |
|  | How often during the past 6 months have you been distracted by, worried about or stressed about the following issue: | Difficult, poorly functioning information systems or applications | |
| Work-family conflict | How often does your job or career interfere with your responsibilities at home, such as cooking, shopping, childcare, home maintenance and repairs? | | |
|  | How often does your job or career keep you spending the amount of time you would like to spend with your family? | | |
| **Job resources** |  |  |  |
| Job control | My job allows me to make a lot of decisions on my own | | |
|  | I have a lot of say about what happens in my job | | |
|  | On my job, I have very little freedom to decide how I do my work (reversed) | | |
| Team climate | We have a ‘we are together’ attitude | | |
|  | People keep each other informed about work-related issues in the team | | |
|  | People feel understood and accepted by each other | | |
|  | We share information generally in the team rather than keeping it to ourselves | | |
| Organizational justice | Everybody is entitled to express their opinions and views in matters that concern them | | |
|  | The procedures at the workplace have been applied consistently | | |
|  | The procedures at the workplace have been free of bias | | |
|  | My supervisor treats me with dignity | | |
|  | My supervisor treats me with respect | | |
|  | My supervisor considers my needs and listens to me | | |
|  | My outcomes reflect the effort I put into my work | | |
|  | My outcomes are appropriate for the work I have completed | | |
